# Effect of petroleum-derived substances on life history traits of bird cherry-oat aphid (*Rhopalosiphum padi* L.) and on the growth and chemical composition of winter wheat

**DOI:** 10.1007/s11356-018-2723-6

**Published:** 2018-07-16

**Authors:** Milena Rusin, Janina Gospodarek, Aleksandra Nadgórska-Socha, Gabriela Barczyk, Elżbieta Boligłowa, Marzena Dabioch

**Affiliations:** 1 Department of Agricultural Environment Protection, University of Agriculture, al. A. Mickiewicza 21, 31-120 Krakow, Poland; 2 Department of Ecology, University of Silesia, Bankowa 9, 40-007 Katowice, Poland; 3 Department of Analytical Chemistry, University of Silesia, Szkolna 7, 40-006 Katowice, Poland

**Keywords:** Petroleum-derived substances *Rhopalosiphum padi* L*.*, Winter wheat, Heavy metals, Macronutrients, Micronutrients

## Abstract

The aim of the study was to determine the effects of various petroleum-derived substances (PDSs), namely petrol, diesel fuel, and spent engine oil, on life history traits of the bird cherry-oat aphid *Rhopalosiphum padi* L., and on the growth and chemical composition of its host plant—winter wheat *Triticum aestivum* L. Each substance was tested separately, using two concentrations (9 and 18 g kg^−1^). Plants were cultivated in both control and contaminated soils. In early October 2013, soil was contaminated and after 1 week, winter wheat seeds, ‘Batuta’ cultivar, were sown. In early June 2014, observations of the effect of petroleum-derived substances on traits of three successive generations of aphids were conducted. Aphids were inoculated separately on leaves using cylindrical cages hermetically closed on both sides. Contamination of aphid occurred through its host plant. Results showed that all of the applied petroleum-derived substances have a generally adverse effect on the developmental parameters in aphids, resulting in the decrease of its fecundity, shortening its average life span, and most often lowering of the population intrinsic growth rate. PDSs caused the limitation of growth in wheat plants; whereas, changes in nutrient contents and heavy metals depended on the part of the plant analysed, the substance applied, and on its dose. The negative relationships between the contents of both some macro-elements (Ca, K, P) and heavy metals (Mn, Cd, Cu, and Zn) and the developmental parameters of particular generations of *R. padi* were observed. The high susceptibility of *R. padi* to the presence of PDSs in the substrate for the host plant should be emphasised—the clear-cut changes in the life span and fecundity, with relatively small changes in the chemical composition of the plant, constitute an evident indication that the developmental parameters of aphids have the potential for the use as bio-indicator to evaluate the state of the environment contaminated by PDSs.

## Introduction

The soil contamination with petroleum-derived substances (PDSs), chiefly associated with the development of petroleum industry, is one of the most important issues in the natural environment (Jørgensen et al. 2000; Ziółkowska and Wyszkowski 2010). PDSs have adverse effects on all the components of the environment, and many of these have carcinogenic and mutagenic effects (Iturbe et al. 2007; Jain et al. 2011). PDSs modify the physical, chemical, and biological properties of soils and, by so doing, can negatively affect the growth and development of cultivated plants (Shirdam et al. 2008; Gbadebo and Adenuga 2012). Penetrating into plants, they move in the intercellular spaces as well as in the vascular system. The particles of carbohydrates cause damage in cell walls, which leads to the leakage of the content of plant tissues, plasmolysis, the deformation of cells, disturbances in the function of conductive tissue, and the dehydration of tissues. In conditions of soil contamination with PDSs, the growth of plant roots is inhibited as well as that of root hairs (Omosun et al. 2008). In cereals, the deposition of PDSs is observed around cell walls and parenchyma impeding water transport. The aboveground parts of plants growing in the conditions of soil contamination with petroleum are characterised by tiny leaves with small assimilation surfaces, visible symptoms of chloroses and necroses, and by thin, short, and poorly branched stems (Wyszkowski and Wyszkowska 2005).

The effects of PDSs on the growth and development of plants and other living things exposed directly to the contact with pollutants are relatively well known, but there is little information available on the topic of indirect effects of these compounds from polluted soil through plants to herbivores. Moreover, the studies to date point to the high usefulness of such traits as fecundity, life span, and other developmental parameters in invertebrates as sensitive indicators of the condition of the environment (Cortet et al. 1999; Gospodarek 2005; Gospodarek and Nadgórska-Socha 2016). Aphids are very dangerous and some of the most common pests of cereals. Both adult individuals and larvae puncture leaves, shoots, flowers, and other vegetative and generative organs, subsequently sucking the juices, which leads to the yellowing, withering, and death of plants. Furthermore, these pests are vector of many dangerous viral diseases (Hansen et al. 2008; Sadeghi et al. 2009). In recent years, as a result of many simplified cultivation practices, as well as with the favourable climatic conditions, an increased significance of the bird cherry-oat aphid (*Rhopalosiphum padi* L.) in farmland cultivars is noted. It pertains to the changes in the development of this pest and with the emergence of new anholocyclic forms which transfer the barley yellow dwarf virus (BYDV) (Hu et al. 2007; Strażyński and Ruszkowska 2015).

The aim of the study was to establish the effect of different concentrations of petroleum-derived substances, such as petrol, diesel fuel, and spent engine oil on the life history traits of three generations of aphids *Rhopalosiphum padi* L. feeding on winter wheat. Furthermore, the effect of above-mentioned substances on the growth of the host plant, content of selected nutrients and heavy metals in its organs was investigated. The research sought to determine whether soil contamination may affect organisms indirectly exposed to pollution and on the further links of the food chain. It was also attempted to determine the aphids’ usefulness as bio-indicator of oil pollution in the environment.

## Materials and methods

### Experimental setup

The experiment was performed in 2014 in pots that could hold 3.8 kg of soil dry mass. In early October 2013, soil (loamy-sand, pH in KCl = 6.12, pH in H_2_O = 6.98, water-holding capacity = 29.5%, total organic C = 0.97%, N content = 0.09%, available K content = 13.00 mg K_2_O 100 g^−1^, available P content = 11.85 mg P_2_O_5_ 100 g^−1^, Pb content = 25.5 mg kg^−1^, Cd content = 0.99 mg kg^−1^, Ni content = 2.19 mg kg^−1^, Zn content = 51.7 mg kg^−1^, Cu content = 5.02 mg kg^−1^, cation exchange capacity: Ca = 3.71 cmol kg^−1^, Mg = 0.37 cmol kg^−1^, K = 0.33 cmol kg^−1^, Na = 0.01 cmol kg^−1^) was collected from uncultivated areas from levels 0–20 cm, finely ground, and subsequently spread to form a thin layer on a foil mat. Prior to contamination with PDSs, basal fertilisation was applied by treating the soil with 0.27 g N (in form of NH_4_NO_3_), 0.14 g P (in the form of KH_2_PO_4_), and 0.21 g K (in the form of KCl) per pot. After thoroughly mixing the soil, it was contaminated with engine oil, diesel fuel, and petrol at two doses: dose I—9 g of PDS per kg of soil dry mass, dose II—18 g of PDS per kg of soil dry mass. Petrol and diesel fuel came from BP petrol station whereas engine oil from Orlen petrol station. Petrol (BP Unleaded 95) is a complex mixture of volatile hydrocarbons containing paraffins, naphthenes, olefins, and aromatic hydrocarbons which contain between C4 and C12 atoms in the molecule (detailed description of the ingredients in Material Safety Data Sheet: http://www.bp.com/content/dam/bp-plus/pl_pl/downloads/PDF/SDS%20benzyna%2095.pdf). Diesel fuel (BP Diesel Fuel) is a mixture of hydrocarbons middle distillates, made between C10 and C28 atoms and may also contain fatty acid methyl ester (FAME) (detailed description in Material Safety Data Sheet: http://www.bp.com/content/dam/bp-plus/pl_pl/downloads/PDF/SDS%20Dieselpdf.pdf). Engine oil (PLATINUM Classic Semisynthetic 10 W-40) is a mixture of mineral and synthetic base oils, enriching additives (detailed description in Material Safety Data Sheet: http://www.orlenoil.pl/_layouts/OrlenOilDownload/Download.ashx?downloadUrl=/PL/NaszaOferta/KartyCharakterystyki/KartyCharakterystyki/1117.pdf?), and it was used for 1 year (in a petrol engine) prior to application in this experiment.

In order to evenly distribute the contaminants, the predetermined amount of each PDS was poured onto a thinly spread layer of soil on a foil mat using a laboratory pipette. The soil was then thoroughly mixed several times and placed in pots. The area of pot was 706.5 cm^2^ (30 cm in diameter). The non-contaminated soil was placed in identical pots and constituted the control treatment. After 1 week, winter wheat (*Triticum aestivum* L.) seeds, ‘Batuta’ cultivar, were sown in each pot at an amount of 15 seeds per pot. After sprouting, the plants were thinned out and ten plants were left in each pot to avoid the competition between germinated seedlings. The pot experiment was performed in quadruplicate.

### *Rhopalosiphum padi* L. traits and population dynamics

Aphids were inoculated on plants in early June using cylindrical cages (12 cm diameter × 20 cm height) made of closely woven airy fabric placed on winter wheat leaves. The cages were attached to the leaves and hermetically closed on both sides in order to avoid the escape of aphids. The investigations were conducted on *R. padi* individuals from the own culture of the Agricultural Environment Protection Department maintained on the same host plant, i.e. winter wheat, Batuta c.v. Three wingless aphid females were placed in each cage and removed completely once they gave birth to the first larvae. One larva was left in each cage. After it reached sexual maturity, its fecundity was determined every day, while newborn larvae were removed each time except the first one, which was transferred immediately to new cage in order to determine the demographic indicators of subsequent generation. One cage was placed on each test plant to investigate the life history of the first generation (40 cages in total for treatment) to ensure the right final number of females to study the aphid life history of *R. padi*. This procedure was applied because some females initially placed on the host plant (using a brush) died before giving birth to the larvae. Cages used to monitor the second and third generation were placed on the subsequent leaves of winter wheat plants, adopting the rule that it should always be on leaves from the same foliage level of the plant in each treatment and generation analysed. Aphid life span and fecundity were assessed for 25 females of each generation and treatment. The population intrinsic growth rate was calculated using the formula developed by Wyatt and White (1977):

1 $$ {r}_m=\left(0.783\cdot In{M}_d\right)/d $$

where

*r*_*m*_: is the population intrinsic growth rate,
*d*: is the duration of pre-reproductive period (from birth to producing the first offspring),
*M*_*d*_: is the mean number of larvae born in the period from *d* to 2*d* days from birth.

The constant value of 0.738 is an approximation of the proportion of the total fecundity produced by a female in the period from *d* to 2*d* days from birth.

### Growth of winter wheat plants

After the experiment was finished, plants were harvested from the pots, and their morphology was evaluated in laboratory conditions (height of plant, length and mass of ear, length and mass of stem, number and mass of grains per ear).

### Chemical composition of plants

In order to determine the nutrients (phosphorus, potassium, calcium, magnesium, iron) and heavy metal (copper, manganese, nickel, lead, zinc, cadmium) concentrations in plant parts, plant material was cleaned of any patches of deposited aphid honeydew and other surface contaminants, washed in tap, next in distilled water. It was then dried at 105 °C for 48 h. A portion of 0.25 g dried plant material was digested with 5 ml of HNO_3_ at 110 °C and then diluted to 25 mL with deionised water. Next, the metal content was measured using flame absorption spectrometry (Unicam 939 Solar) (Azcue and Mudroch 1994; Nadgórska-Socha et al. 2013). The quality of the analytical procedure was checked using a reference material (Certified Reference Material CTA-OTL-1 Oriental Tobacco Leaves) with the same quantities of samples. Nitrogen and sulphur contents were determined in a Variomax CNS analyser. The analyses were performed separately for roots, and for shoots and leaves (defined jointly as aboveground parts), and for grain. The following indicators were calculated: K/(Ca + Mg), Ca/Mg, N/S, representing ratios between selected nutrients in plants.

### Statistical analysis

The obtained results were analysed and checked for normality (Shapiro–Wilk test with Lilliefors correction) and equality of variance (Levene’s test) and when necessary, the data were log transformed. The significance of differences between the means were tested by one-factor variance analysis (STATISTICA 10.0 software), and the means were differentiated by Fisher’s LSD test at *p* < 0.05.

Multiple regression equations were derived to determine which of accumulated heavy metal and nutrient contents influenced aphid traits. The method of stepwise forward regression was applied. The equations concerned the relationships between life span, fecundity, as well as intrinsic growth rate and the estimated and accumulated nutrients (calcium, potassium, iron, magnesium) and heavy metals: copper, manganese, nickel, lead, zinc, cadmium. Regression equations were derived for three generations of aphids.

CANOCO 4.5 was used to carry out Principal Component Analysis (PCA) and Redundancy Analysis (RDA) (Ter Braak and Šmilauer 2002). Principal Component Analysis assessed the relationships between plant composition and PDS contamination. Redundancy Analysis assessed the relationships between plant composition, PDS contamination, and aphid traits.

## Results

### Life cycle traits of *Rhopalosiphum padi* L.

**Fig. 1:**
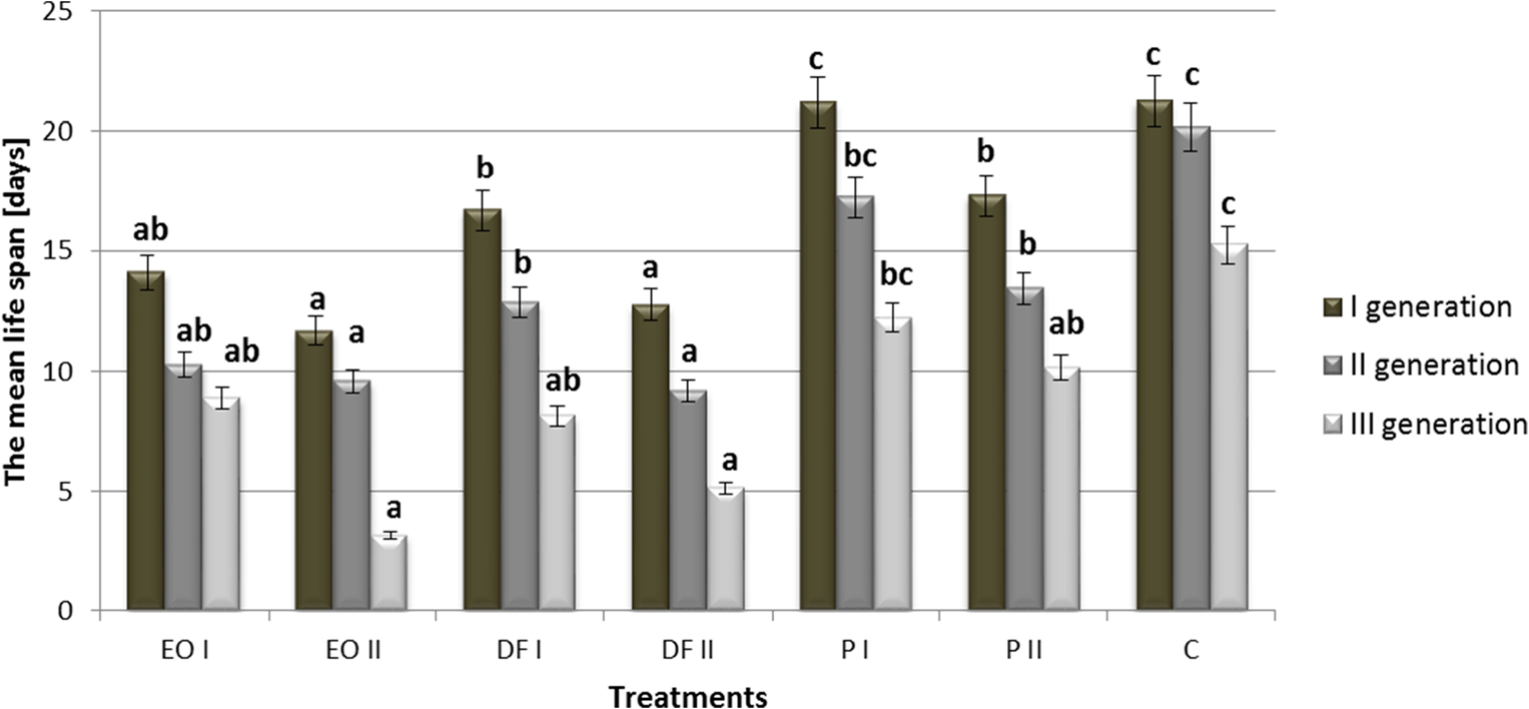
The effect of petroleum-derived substances on mean life span of *Rhopalosiphum padi* L. (days). C control soil, P soil contaminated with petrol, EO soil contaminated with engine oil, DF soil contaminated with diesel fuel, I, II doses of pollutants. Values marked by different letters for each generation are statistically different (*p* < 0.05)

Compared with the control plants, both engine oil and diesel fuel in two doses applied resulted in a significant shortening of life span in three studied generations of *R. padi* feeding on the plants growing on contaminated soil (Fig. 1). Similar regularities were also noted in the case of an object polluted with petrol, albeit only after the higher dose of this substance (18 g kg^−1^) had been applied. In line with the increase in the content of particular xenobiotics in soil, the negative effects of the analysed parameter also intensified, and the statistically significant differences between objects with particular doses of PDSs were noted in the first- and second- generations after applying diesel fuel, and in the first generation after applying petrol. In all analysed objects (including the control), the life spans of aphids decreased with time and with the emerging generation of the pest.

**Fig. 2:**
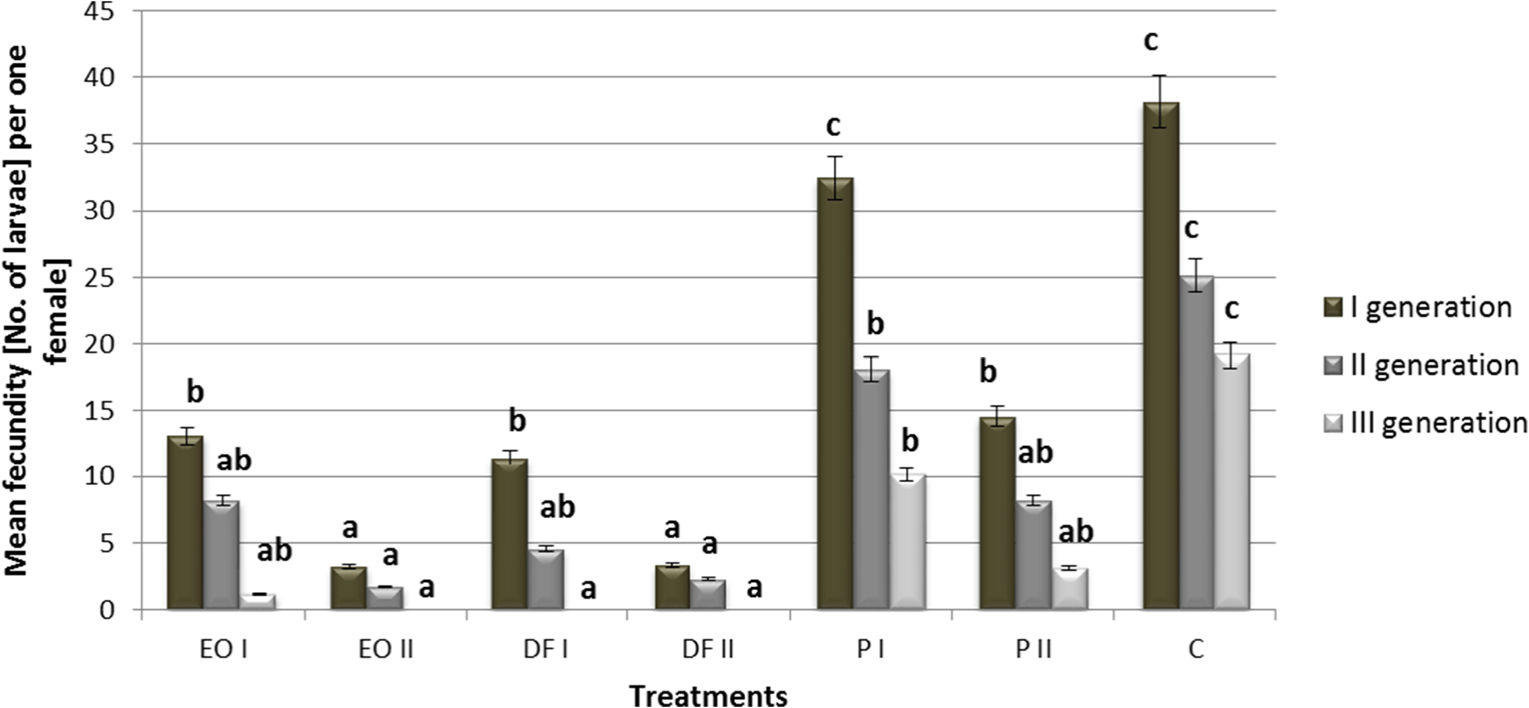
The effect of petroleum-derived substances on mean fecundity of *Rhopalosiphum padi* L. (No. of larvae per one female). Symbols as in Fig. 1. Values marked by different letters for each generation are statistically different (*p* < 0.05)

Applying PDSs resulted in a significant reduction in fecundity in all generations of *R. padi* females in both doses used (except for the lower dose of petrol in the first generation of the pest) (Fig. 2). The most detrimental effects on the analysed parameter were exerted by engine oil and diesel fuel in the dose of 18 g kg^−1^, which caused the reduction in the fecundity of the first- and second-generation females by more than 90% and completely inhibited the process of giving birth to larvae in the case of third generation. Similarly as in the previously analysed case, the fecundity of aphids decreased in the subsequent generations.

The duration of pre-reproductive period (*d*) *R. padi* fell in the range from 9.5 to 11.1 days for the first generation, from 9.0 to 10.2 days for the second generation, and from 8.9 to 9.8 days for the third generation (Table 1). Higher doses of PDSs (18 g kg^−1^) resulted in a significant lengthening of the pre-production period in first-generation females; however, no such relationships were noted in the remaining generation of the pest. Both doses of diesel fuel used, as well as a dose of engine oil amounting to 18 g kg^−1^, completely inhibited the process of giving birth to larvae in the third-generation females.

**Table 1:** The effect of petroleum-derived substances on some biological parameters (*d* duration of pre-reproductive period, *M*_*d*_ mean number of larvae born in time = *d*) of *Rhopalosiphum padi* L.

Details	*d*			*M*_*d*_		
Generation	First	Second	Third	First	Second	Third
EO I	10.2^ab*^	9.6^ab^	8.9^a^	13.1^b^	8.1^ab^	1.8^a^
EO II	10.7^c^	9.4^a^	–	3.2^a^	1.7^a^	0^a^
DF I	9.8^a^	10.2^b^	–	11.5^b^	4.5^a^	0^a^
DF II	11.1^c^	9.0^a^	–	3.3^a^	2.3^a^	0^a^
P I	10.0^a^	9.5^ab^	9.6^a^	26.8^c^	18.2^c^	10.1^b^
P II	10.3^b^	10.1^b^	9.8^a^	14.6^b^	8.2^b^	3.1^a^
C	9.5^a^	9.8^ab^	9.8^a^	33.1^c^	22.8^c^	19.2^c^

*Means in columns marked with the same letters do not differ significantly according to LSD test at *p* < 0.05. Symbols as in Fig. 1

The average number of larvae born by females in the period of reproduction equal to *d* (*M*_*d*_) in all generations was the highest in the control object (Table 1). Engine oil and diesel fuel in both doses applied, as well as the higher dose of petrol, resulted in a significant decrease in the value of *M*_*d*_ indicator in all generations of *R. padi*. In the third generation of *R. padi,* the value of *M*_*d*_ was also significantly lower after the application of a lower dose of petrol.

**Fig. 3:**
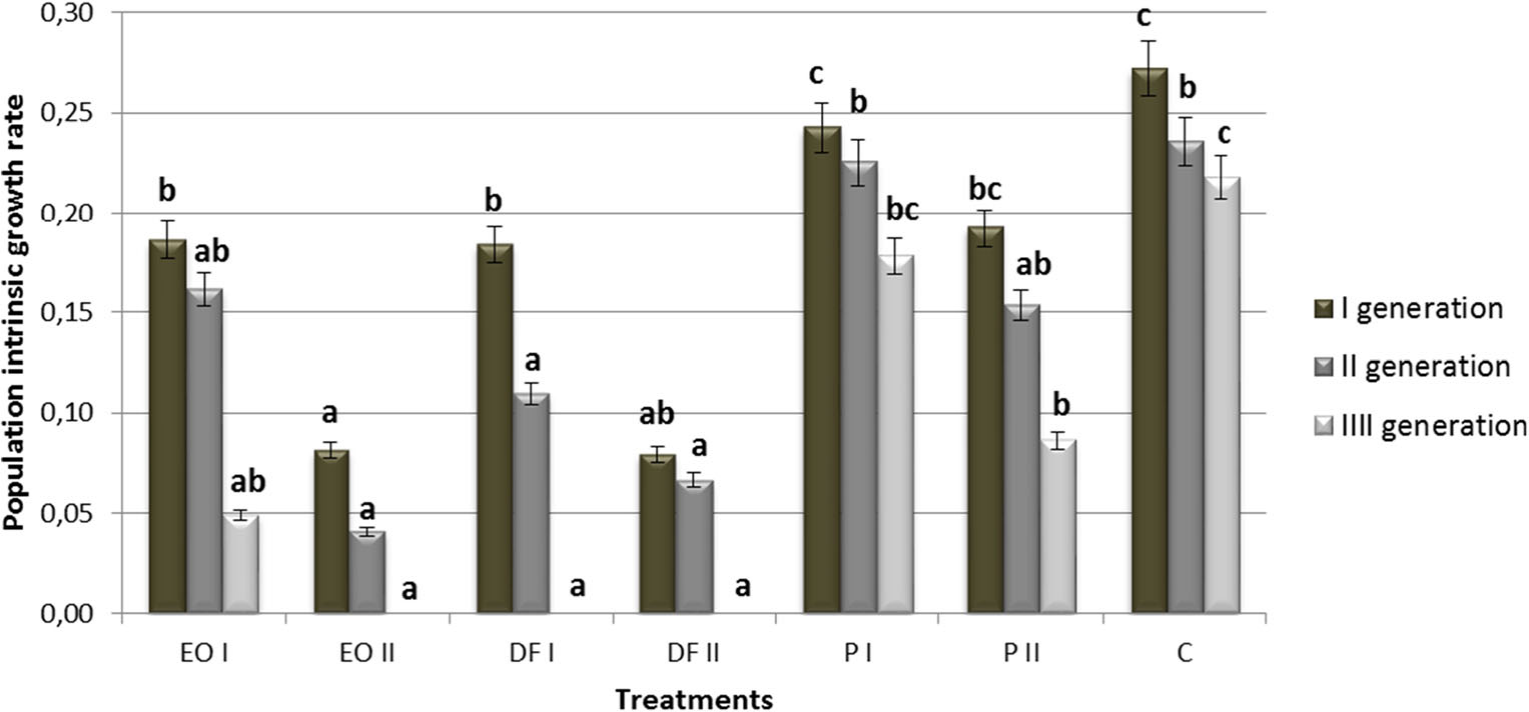
The effect of petroleum-derived substances on population intrinsic growth rate (*r*_*m*_) of *Rhopalosiphum padi* L. Symbols as in Fig. 1. Values marked by different letters for each generation are statistically different (*p* < 0.05)

The population intrinsic growth rate (*r*_*m*_) reached its highest values in the first generation of *R. padi* and it decreased in subsequent generations, and in the case of objects contaminated with diesel fuel in both doses, and with engine oil in 18 g kg^−1^ dose, assumed zero value for the third generation of pest (Fig. 3). Compared with the control object, diesel fuel in both doses applied caused a significant decrease in the population intrinsic growth rate in all generation of the aphid. In the case of engine oil, a similar relationship was noted, except for the second generation and the lower dose of this xenobiotic. In turn, petrol had generally no significant effects upon the analysed parameter (except for the third generation and the higher dose of this substance).

### Growth of winter wheat plants

Irrespective of the dose, the contamination of soil with diesel fuel affected the growth of winter wheat plants, resulting in the significant decrease in plant height, ear length, as well as the length and mass of stem, and the number and mass of grains in the ear (Table 2). Similar regularities were also observed in the object contaminated with the higher dose of engine oil. The plants growing on soil contaminated with petrol in 18 g kg^−1^ dose had significantly shorter stems than that in control plants, as well as a lower number and mass of grains in the ear. The lower dose of petrol decreased only the number of grains per ear. With the increased dose of the xenobiotic applied, its negative effect on the analysed morphological parameters of plants had most often increased as well.

**Table 2:** The effect of petroleum-derived substances on the growth of *Triticum aestivum* L.

Details	Height of plant (cm)	Length of ear (cm)	Mass of ear (g)	Length of stem (cm)	Mass of stem (g)	Number of grains per ear (pcs.)	Mass of grains per ear (pcs.)
EO I	46.50^b*^	5.43^bc^	0,80^a^	41.07^b^	0.80^bc^	14.30^ab^	0.52^bc^
EO II	43.44^ab^	4.25^a^	0.74^a^	39.20^ab^	0.62^ab^	11.00^a^	0.41^abc^
DF I	41.70^a^	4.77^ab^	0,57^a^	36.93^a^	0.59^ab^	10.50^a^	0.31^a^
DF II	43.97^ab^	4.27^a^	0.64^a^	39.70^ab^	0.52^a^	10.90^a^	0.38^ab^
P I	51.93^c^	6,00^c^	0.74^a^	45.93^c^	1.01^c^	12.70^a^	0.45^abc^
P II	47.67^b^	6.02^c^	0.63^a^	41.65^b^	0.90^c^	13.17^a^	0.34^ab^
C	53.70^c^	6.10^c^	0.84^a^	47.60^c^	0.80^bc^	18.33^b^	0.56^c^

*Means in columns marked with the same letters do not differ significantly according to LSD test at *p* < 0.05. Symbols as in Fig. 1

### Content of selected macrocomponents and heavy metals in plants

The effects of PDSs upon the content of nutrients in plants was variable and dependent on the kind of analysed nutrient, dose, and the kind of the substances applied, as well as on the part of plant (Table 3). All substances applied in the experiment generally led to a significant increase in nitrogen content in the roots and grain of winter wheat; whereas, the application of petrol also resulted in increases in the contents of this macro-component in the aboveground parts of plants (i.e. in shoots and leaves). Engine oil brought about the significant increases in the contents of P and Ca in the aboveground parts of winter wheat plants as well the increase in Fe content in grain. The higher dose of the substance also led to the increases in K, Mg, and Fe contents in the aboveground parts, and to S, P, and K contents in roots; however, the dose of 9 g kg^−1^ resulted in the decreases of P, Mg, and Fe contents in plant roots. Diesel oil contributed to the increase in Ca content in the aboveground parts and roots of winter wheat, and to the increases in S and P contents in roots. On the other hand, it decreased the Fe content in roots, and—in the lower dose—also led to the decreases in S, K, and Ca contents in grain; whereas, in the dose of 18 g kg^−1^ led to the decrease in S and Fe contents in the aboveground parts of plants. The lower dose of petrol resulted in the increases of S, P, K, and Ca contents in the aboveground parts and roots; whereas, the dose of this substance amounting to 18 g kg^−1^ contributed to the decreases in the contents of all analysed nutrients (except for N) in roots, and to the decrease in Ca and Mg contents in aboveground parts.

**Table 3:** The effect of petroleum-derived substances on content of selected nutrients in *Triticum aestivum* L. (g kg^−1^)

Details	N	S	P	K	Ca	Mg	Fe
Aboveground parts							
EO I	6.56^bc*^	3.74^bc^	6.94^c^	12.36^bc^	2.80^c^	0.66^bc^	0.73^a^
EO II	5.60^a^	3.73^bc^	6.85^c^	14.68^d^	3.63^e^	0.75^d^	1.61^c^
DF I	7.13^c^	3.32^b^	4.21^b^	9.56^a^	3.17^cd^	0.67^bc^	1.33^b^
DF II	6.01^ab^	2.30^a^	2.15^a^	10.20^ab^	3.42^de^	0.62^b^	0.76^a^
P I	10.95^d^	7.19^d^	8,56^e^	14.25^cd^	2.91^c^	0.72^cd^	0.75^a^
P II	11.63^d^	4.01^c^	7.79^d^	9.92^a^	1.54^a^	0.53^a^	0.66^a^
C	6.66^bc^	3.76^bc^	4,25^b^	11.33^ab^	2.15^b^	0.65^bc^	0.71^a^
Roots							
EO I	9.58^ab^	2.46^b^	2.26^b^	1.29^bc^	2.73^b^	0.51^b^	2.34^a^
EO II	12.14^cd^	3.39^c^	3.79^de^	1.48^c^	2.78^b^	0.85^cd^	3.53^b^
DF I	12.88^d^	3.69^cd^	3.50^d^	1.38^bc^	3.12^c^	0.87^cd^	3.50^b^
DF II	12.11^cd^	3.96^d^	3.96^e^	1.32^bc^	3.25^c^	0.76^c^	3.17^b^
P I	10.72^bc^	3.49^c^	3.64^d^	2.26^d^	3.17^c^	0.89^d^	4.24^c^
P II	10.73^bc^	1.45^a^	1.27^a^	0.37^a^	1.42^a^	0.22^a^	2.18^a^
C	8.34^a^	2.17^b^	2.63^c^	1.25^b^	2.57^b^	0.87^cd^	4.95^d^
Grain							
EO I	24.84^c^	4.46^b^	12.88^cd^	3.10^bc^	0.23^b^	1.37^d^	0.12^b^
EO II	23.75^b^	4.22^ab^	12.47^bc^	3.12^bc^	0.22^ab^	1.37^d^	0.42^c^
DF I	23.67^b^	3.95^a^	11.49^ab^	3.01^b^	0.21^ab^	1.19^ab^	0.05^a^
DF II	25.94^d^	4.50^b^	11.00^a^	2.62^a^	0.32^c^	1.22^abc^	0.04^a^
P I	23.60^b^	4.08^ab^	12.00^abc^	3.06^bc^	0.18^a^	1.11^a^	0.04^a^
P II	30.23^e^	5.43^c^	13.75^d^	3.48^d^	0.35^c^	1.33^cd^	0.06^a^
C	20.94^a^	4.49^b^	11.97^abc^	3.36^cd^	0.36^c^	1.25^bcd^	0.05^a^

*Means in columns for each organ of plant marked with the same letters do not differ significantly according to LSD test at *p* < 0.05. Symbols as in Fig. 1

PDSs applied in the experiment had generally caused an increase in the value of Ca/Mg ratio in aboveground parts of winter wheat, as well as in roots, but also decreased its value in the grain of plants (Table 4). Engine oil was applied at the higher dose, and the diesel fuel in both doses applied led to the drop in the value of K/(Ca + Mg) ratio in aboveground parts, although they most often did not affect the value of the analysed ratio in other organs of plants. All xenobiotics caused the increased in the value of N/S ratio in the grain of plants, while diesel fuel and petrol in higher doses also caused the increase in its value in aboveground parts.

**Table 4:** The effect of petroleum-derived substances on the ratio of nutrients in *Triticum aestivum* L.

Details	K/(Ca + Mg)	Ca/Mg	N/S
Aboveground parts			
EO I	3.58^bc*^	4.27^b^	1.86^ab^
EO II	3.34^b^	4.82^c^	1.56^a^
DF I	2.50^a^	4.74^c^	2.12^b^
DF II	2.53^a^	5.54^d^	2.79^c^
P I	3.87^c^	4.06^b^	1.56^a^
P II	4.81^d^	2.94^a^	2.90^c^
C	4.05^c^	3.34^a^	1.72^a^
Roots			
EO I	0.40^c^	3.66^b^	3.94^a^
EO II	0.41^c^	4.20^c^	3.57^a^
DF I	0.34^bc^	5.38^d^	3.39^a^
DF II	0.33^b^	6.55^e^	3.06^a^
P I	0.56^d^	3.27^ab^	3.25^a^
P II	0.23^a^	3.56^b^	7.41^b^
C	0.36^bc^	2.97^a^	3.84^a^
Grain			
EO I	1.93^b^	0.17^a^	5.51^b^
EO II	1.95^bc^	0.17^a^	5.73^b^
DF I	2.17^cd^	0.18^a^	8.58^b^
DF II	1.70^a^	0.26^b^	6.04^b^
P I	2.38^d^	0.16^a^	6.06^b^
P II	2.07^bc^	0.26^b^	5.69^b^
C	2.08^bc^	0.29^b^	4.62^a^

*Means in columns for each organ of plant marked with the same letters do not differ significantly according to LSD test at *p* < 0.05. Symbols as in Fig. 1

As in the case of macro-components, the contents of heavy metals in plants caused by the presence of PDSs in soil was variable (Table 5). In general, the applied substances caused the increase in Mn content in aboveground parts and roots of winter wheat, but the decrease in the content of this metal in grain. Engine oil in both doses applied contributed to the increases in Pb and Ni contents in grain and to Pb content in the aboveground parts of plants. The dose of 9 g kg^−1^ of this substance led to the decreases in the contents of Cu, Ni, and Pb in plant roots; whereas, the higher dose led to the increase in Cu content in aboveground parts and in grain and to the increase in Zn content in roots. Diesel fuel caused the increase in Zn content in roots but the decrease of its content in grain. The lower dose of this substance led to the increase in Cu and Pb content in aboveground parts, while the higher dose led to the increased content of Cu and Ni in roots and of Pb in grain of winter wheat. Petrol in the dose of 18 g kg^−1^ generally caused the decrease in the content of analysed heavy metals (except for Mn and Cd) in plant roots, but also the increase in Ni and Zn contents in grain.

**Table 5:** The effect of petroleum-derived substances on content of selected heavy metals in *Triticum aestivum* L. (mg kg^−1^)

Details	Cu	Mn	Ni	Pb	Zn	Cd
Aboveground parts						
EO I	10.88^bcd*^	181.72^a^	1.57^ab^	9.73^c^	361.61^a^	3.22^b^
EO II	14.18^e^	380.59^d^	2.42^c^	9.10^c^	302.84^a^	2.23^a^
DF I	12.16^d^	203.14^a^	2.05^bc^	9.38^c^	394.88^a^	2.49^a^
DF II	6.95^a^	240.53^b^	1.56^ab^	7.07^b^	303.13^a^	1.91^a^
P I	11.33^cd^	265.98^c^	1.41^a^	12.17^d^	299.42^a^	3.78^b^
P II	8.87^ab^	250.16^bc^	1.64^ab^	4.03^a^	373.02^a^	2.42^a^
C	9.65^bc^	184.89^a^	2.04^bc^	5.82^b^	310.65^a^	2.10^a^
Roots						
EO I	15.38^b^	689.01^c^	3.06^b^	15.73^b^	417.70^b^	0.82^a^
EO II	20.82^cd^	435.85^b^	4.11^bc^	23.61^c^	677.14^c^	1.39^b^
DF I	21.97^d^	889.55^d^	4.82^c^	25.28^c^	705.52^c^	0.89^ab^
DF II	36.94^e^	1107.46^e^	7.04^d^	32.06^c^	974.05^d^	1.14^ab^
P I	18.96^c^	407.42^ab^	4.38^c^	27.95^cd^	411.56^b^	2.31^c^
P II	9.71^a^	874.55^d^	1.43^a^	8.68^a^	130.32^a^	1.93^c^
C	20.77^cd^	308.17^a^	4.79^c^	23.58^c^	427.06^b^	1.18^ab^
Grain						
EO I	9.56^cd^	67.12^ab^	0.55^c^	1.32^c^	102.22^d^	0.12^b^
EO II	10.71^d^	80.50^bc^	0.77^d^	1.67^c^	89.50^bc^	0.01^a^
DF I	7.62^ab^	49.90^a^	0.08^b^	0.64^b^	59.63^a^	0.02^a^
DF II	6.31^a^	49.35^a^	0.01^a^	1.61^c^	63.34^a^	0.02^a^
P I	7.48^ab^	49.86^a^	0.52^c^	0.48^ab^	78.05^b^	0.35^c^
P II	9.66^cd^	50.93^a^	0.37^c^	0.25^a^	100.04^cd^	0.13^b^
C	8.35^bc^	94.61^c^	0.14^b^	0.83^b^	79.32^b^	0.10^b^

*Means in columns for each organ of plant marked with the same letters do not differ significantly according to LSD test at *p* < 0.05. Symbols as in Fig. 1

### Relationships between *R. padi* traits, soil contamination with PDSs, and winter wheat chemical composition

**Fig. 4:**
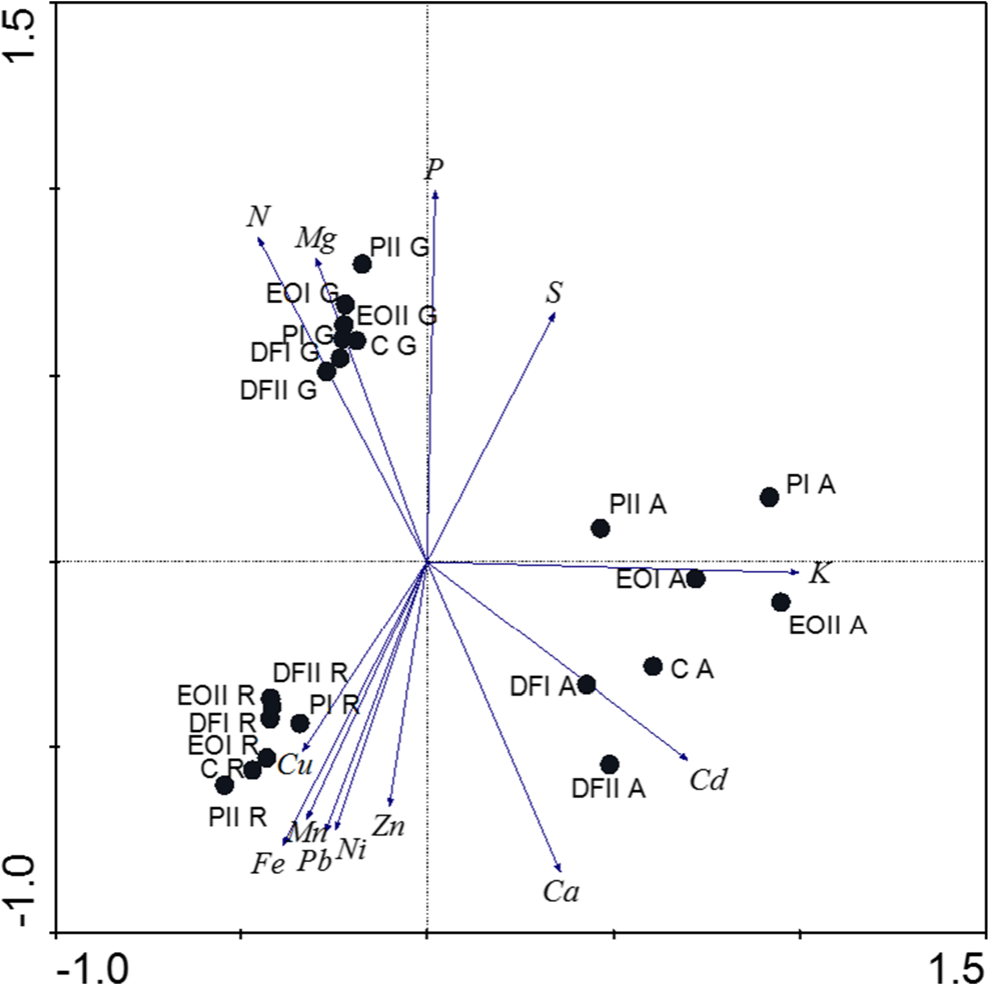
Principal component analysis of element levels in *Triticum aestivum* L. organs, and soil contamination with petroleum-derived substances. Symbols as in Fig. 1. A aboveground parts, R roots, G grain

**Fig. 5:**
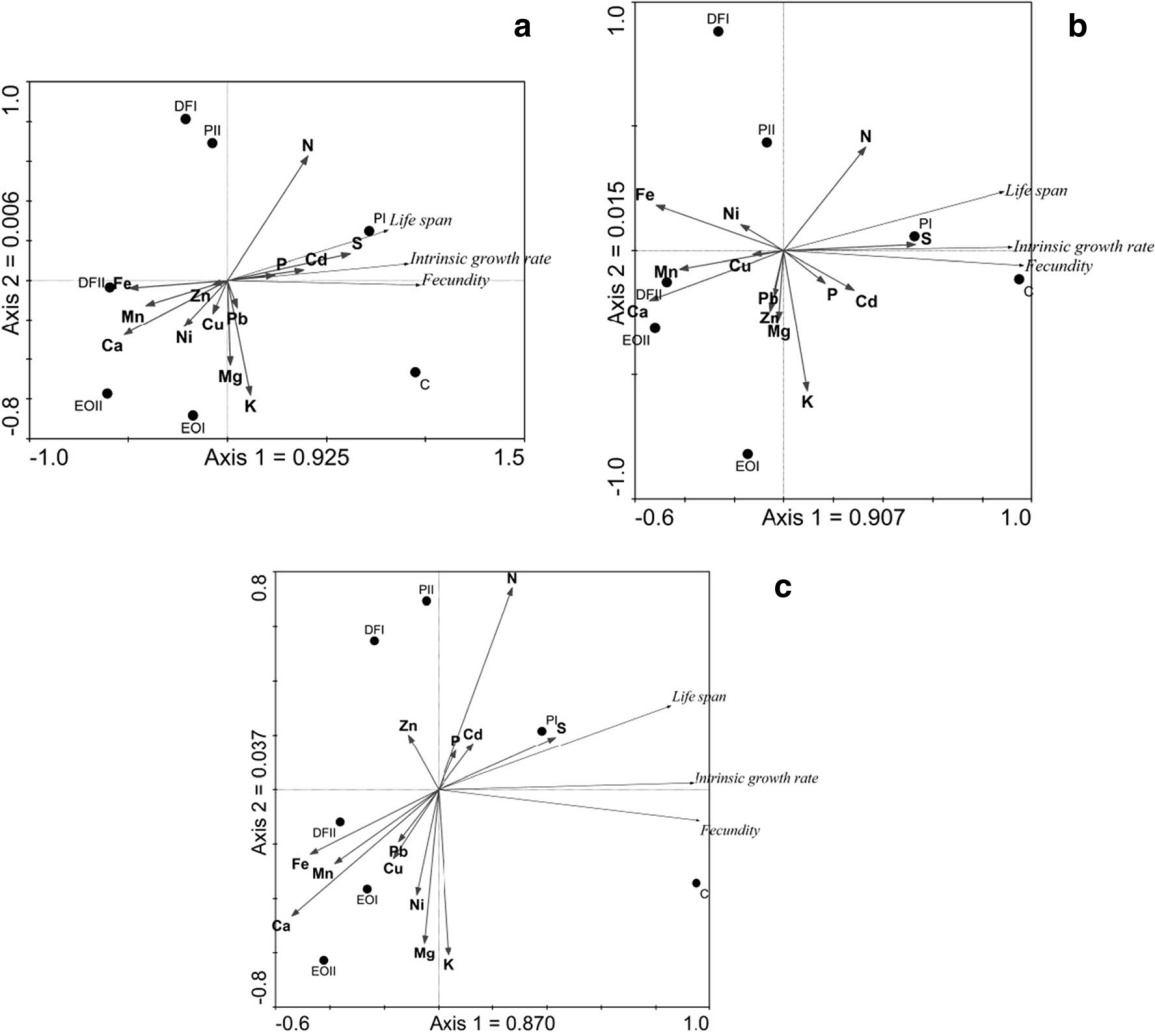
Redundancy analysis of element levels in *Triticum aestivum* L. aboveground parts grown on soil contaminated with petroleum-derived substances and aphid traits (**a** first generation, **b** second generation, **c** third generation). Symbols as in Fig. 1

The first and second ordination axes explain the similar percentage of variation, dividing the samples into three sets (A, G, R). The potassium content was the most correlated with the first ordination axis whereas the Mg, P, and Ca contents—with the second ordination axis. The highest Mg concentrations were found in G samples. In turn, the highest contents of Cu, Mn, Fe, Pb, Ni, and Zn were found in R samples (Fig. 4). Multiple regressions revealed that Mn and Cd had negative effects on life span, fecundity, and the intrinsic growth rate of aphids in the first generation of aphids (Table 6). In the second generation, Ca had an adverse effect on the studied parameters, and Cu and K also had adverse effects on the fecundity and intrinsic growth rate of aphids. The developmental parameters of the third generation of aphids were adversely affected by Ca, while additionally, the negative effect was exerted by Zn on the life span and fecundity. It was also the P content in plants which had the adverse effect on the fecundity and the intrinsic growth rate in this generation. The above relationships were also confirmed by RDA analysis (Fig. 5).

**Table 6:** Multiple regression equations (*p* < 0.05)

First generation	*R* ^2^
Life span = 26.31 + 1.47(S) − 0.77(Mn) − 1.02(Cd) + 0.13(Cu) (2.54) (0.18) (0.12) (0.19) (0.11)	0.72
Fecundity = 60.74 + 1.71 (S) − 1.09 (Mn) − 1.39(Cd) + 0.28 (K) + 0.29 (P) − 0.25 (N) (11.99) (0.17) (0.08) (0.09) (0.18) (0.12) (0.17)	0.95
Intrinsic growth rate = 0.04 + 1.14(S) − 0.61(Mn) - 0.36 (Cd) + 0.60(Ni) − 0.47(Fe) (0.08) (0.15) (0.10) (0.18) (0.16) (0.17)	0.867
Second generation	
Life span = 88.43–1.13(Ca) − 1.22(Zn) − 0.85(Pb) − 0.56(N) + 1.24(Cd) (7.29) (0.34) (0.20) (0.78) (0.17) (0.66)	0.845
Fecundity = − 63.11 − 1.72(Ca) + 2.41(Mg) − 0.99(Cu) − 0.46(K) (4.68) (0.07) (0.13) (0.07) (0.07)	0.94
Intrinsic growth rate = − 0.46 − 1.54(Ca) + 2.40(Mg) − 1.09(Cu) − 0.75(K) + 0.40(P) (0.06) (0.10) (0.21) (0.12) (0.16) (0.12)	0.90
Third generation	
Life span = 59.12 − 1.16(Ca) − 0.77(Zn) + 0.39(Pb) − 0.48(Fe) + 0.30(Ni) (8.28) (0.14) (0.10) (0.11) (0.20) (0.30)	0.83
Fecundity = − 22.73 − 1.76(Ca) − 0.17(Zn) + 1.78(Mg) − 0.67(Cu) − 0.39(P) (10.14) (0.08) (0.08) (0.13) (0.08) (0.07)	0.94
Intrinsic growth rate = 0.39 + 1.73(S) − 0.75(Ca) − 0.88(N) − 0.61(P) − 0.42(Pb) (0.03) (0.12) (0.10) (0.09) (0.07) (0.12)	0.96

## Discussion

Bird cherry (*Prunus padus*) is the primary host of *R. padi*. Females lay eggs in autumn in the narrow gap between the axillary buds and the stem. After egg hatch, the newly emerged nymphs move to the unfurling bird cherry leaves where they feed and develop. Under favourable conditions, the nymphs develop rapidly into very large light-green mature fundatrices and give rise to a second, wax-covered, generation. Both crowding and poor nutrition induce the appearance of winged emigrants that in May migrate to cereals and grasses and start feeding. In autumn, short day-length induces the appearance of gynoparae on summer hosts. These winged females migrate to bird cherry where senescing leaves provide the aphids with a rich source of food. There they give birth to apterous oviparae. Short day length also induces the somewhat later appearance of winged males on grasses, which migrate to bird cherry where they mate with the oviparae (Delmotte et al. 2002; Jiménez-Martínez et al. 2004). In our experiment, only asexual reproduction of *R. padi* was investigated. However, given that on cereals can develop to 12 asexual generations, this may involve significant implications both for the whole pest population in a given area, and for its natural enemies.

The average fecundity of wingless females of *R. padi* on wheat in uncontaminated conditions ranges to 40 larvae but depends on the cultivars of wheat and the generation of pests. Aphid life span is about 28 days (Aqueel and Leather 2011). In our experiment, analysed fecundity of aphids was at a similar level, while life span was slightly lower. All the PDSs applied in the presented experiment (except for lower dose of petrol) adversely affected the developmental parameters of the bird cherry-oat aphid, resulting in the decreased fecundity, shortening of the average life span, as well as decreasing its intrinsic growth rate in general. In the available publications, there is little information on the effects of soil contamination with PDSs upon the developmental parameters of herbivores. Our earlier studies (Rusin et al. 2017) demonstrated that the contamination of soil with PDSs had a negative impact on the developmental parameters of black bean aphid *Aphis fabae* Scop., which corresponds to the results obtained in the presented experiment involving the bird cherry-oat aphid. In the aforementioned experiments, the same PDSs used in the same doses caused the significant shortening of life span in three generations of *A. fabae* feeding upon the plants growing on contaminated soil, by approx. 30%. In the presented experiment, the higher doses of engine oil and diesel fuel brought about the shortening of the life span of the first and second generation of *R. padi* aphids by approx. 50%, and in the third generation—by approx. 70%. In both experiments, the oils had the most adverse effect on the intrinsic growth rate and fecundity of aphids, and their higher doses had completely inhibited the process of giving birth to larvae by the third-generation females. Along with the increase of the concentrations of PDSs in soil, their adverse effects towards the studied pests also increased. The studies on the bionomics of *A. fabae* on broad bean growing on the soil contaminated by the same PDSs but in lower dose (6 g kg^−1^ of soil) showed the fecundity in the first and second generations lowered by approx. 25%, whereas the life span did not change. All the PDSs, however, caused the significant lowering of the value of intrinsic growth rate in the second generation of *A. fabae* (Gospodarek and Nadgórska-Socha 2016). Because of the above, it seems that *R. padi* is characterised by the higher sensitivity to the presence of PDSs in soil than *A. fabae.*

The changes in the bionomics of bird cherry-oat aphid could be the result of the changes in the nutritive value of host plants under the effect of PDSs, as well as of the presence of harmful substances in tissues resulting from their transfer from contaminated soil. The PDSs contribute to the weakening the growth and development of cultivated plants, and they also modify the contents of macro- and micro-components in these plants, which can lead to the worsening in the quality of fodder for the herbivores (i.e. via decreased content of protein and chlorophyll) (Nadgórska-Socha et al. 2005). Aphids feed by piercing the phloem of their food plant and are very sensitive to changes in plant quality (Omacini et al. 2001). On high quality plants, the aphids produced the most offspring and were characterised by a higher population intrinsic growth rate (Stadler et al. 2002).

In the presented experiment, the majority of PDSs used had adversely affected the growth of winter wheat; however, that effect was not that obvious as in the case of broad bean in our earlier experiment when the same doses of PDSs had been used (Rusin et al. 2017). In the case of broad bean, the oils caused almost a twofold decrease in the mass and length of stems, as well as in the number and mass of leaves. In the case of winter wheat, we noted the shortening of the length of stem by approx. 18% and of the length of ear by more than 23%. It suggests somewhat higher resistance of winter wheat to this type of soil-related factor. Despite this fact, as we mentioned earlier, the adverse effect of PDSs towards the development of *R. padi* on wheat was stronger than that on the development of *A. fabae* on broad bean. It could stem from the species specifics of the herbivore. The high sensitivity of *R. padi* to adverse factors derived from host plant was confirmed by Hale et al. (2003). These authors demonstrated a significant decrease in the intrinsic growth rate of the population of this aphid species on grasses resulting from the worsened morphological parameters (such as growth or leaf number) under the effect of drought. The adverse effect of PDSs upon the growth and development of cultivated plants was demonstrated by a number of authors (Akinola et al. 2004; Wyszkowski et al. 2004; Agbogidi et al. 2006; Liste and Felgentreu 2006; Agbogidi et al. 2007; Njoku et al. 2008; Njoku et al. 2012; Osuagwu et al. 2013; Lopes and Piedade 2014; Rusin et al. 2015). This effect could be a result of disturbances in the intake of essential macro- and micro-elements such as magnesium, iron, boron, and manganese, as well as of water (Odjegba and Atebe 2007). Heavy metals, polycyclic aromatic hydrocarbons, and other chemical additives present in PDSs also can inhibit plant growth (Nwaichi et al. 2014). The PDSs can also block the transportation of substances in plant cells that can contribute to the limited development of vegetative and generative organs (Osuagwu et al. 2013).

PDSs modify the content of nutrients in plants (Wyszkowski et al. 2004, Moubasher et al. 2015, Rusin et al. 2015), with particular species, or even varieties of plants demonstrating different reactions in this respect (Dimitrov and Mitov 1998; Wyszkowski and Ziółkowska 2009). In the presented experiment, PDSs caused the changes in the content of macroelements depending both on the kind of PDS, its dose, the part of the plant, as well as on the analysed element. Considering the aboveground parts, which are the places where the aphids feed, we more often dealt with the increases in the content of macroelements than with their decreases. Similar increases in the content of some macro- and micro-elements in cereals under the effect of the content of engine oil were also demonstrated by Wyszkowski and Wyszkowska (2005) and by Wyszkowski and Ziółkowska (2009).

Nitrogen is considered to be an element whose content in tissues is usually strongly, positively correlated with developmental parameters in different aphid species (Jansson and Ekbom 2002; Davies et al. 2004; Naluyange et al. 2014). However, Aqueel and Leather (2011) demonstrated that increasing nitrogen fertilisation, which contributed to the increased nitrogen content in plants, generally did not significantly affect the fecundity and life span of *R. padi* feeding on wheat, and that it was—to a major extent—dependent on the variety of host plant. Again, in the experiment presented in our study, no beneficial effect of increased nitrogen content in the aboveground parts of wheat plants brought about by the presence of petrol in substrate was noted on the developmental parameters of bird cherry-oat aphid. Moon et al. (1995) also reported that varying nitrogen levels did not affect the duration of pre-reproductive period, reproductive period, and the longevity of another aphid species feeding on wheat—*Diuraphis noxia* (M.).

Engine oil and petrol contributed to the elevated contents of phosphorus in the aboveground parts of wheat plants. The data pertaining to the effect of this element on sucking insects is divergent. In their review, Waring and Cobb (1992) stated that phosphorus most often failed to affect sucking insects (48% of studies they analysed) or affect them positively (in approx. 38% of studies). Later studies (Pitan et al. 2000), however, indicate that this element can also exert the effect in the form of limiting the density of sucking insects. Both the RDA analysis and multiple regression performed showed the adverse effect of P on the fecundity and intrinsic growth rate of the third generation of aphids.

The content of potassium increased under the effect of the presence of a higher dose of engine oil and under the effect of a lower dose of petrol. Myers and Gratton (2006) as well as Myers et al. (2004) demonstrated that *Aphis glycines* foraging on the leaves of potassium-deficient soya had the higher rate of population growth and the higher fecundity compared with the aphids foraging on plants with a lower content of this nutrient, which could partly explain the effect of the aforementioned petroleum derivatives on the fecundity of aphids in our experiment.

Almost all of applied PDSs caused the increases in Ca content in aboveground parts of plants. The adverse effect of Ca towards the developmental parameters of the second and third generation of aphids was also shown by multiple regressions. This element used for liming the soil brought about changes in aphid populations depending on the dose applied, with the higher dose (determined in line with 1.5 hydrolytic acidity) limiting the development of the *Aphis fabae* Scop. aphid population on faba bean (Sądej and Sądej 2001).

Available scientific literature lacks information concerning the effect of other analysed nutrients content on the life history traits of aphids.

Besides the changes in the contents of macroelements, their mutual relationships are also very important from the viewpoint of the nutritive value of the host plant. An optimum value of K/(Ca + Mg) ratio for plants to grow and develop should range within 1.6/1–2.1/1 (Matraszek et al. 2015). In our study, the value of the parameter was very different, depending on the analysed part of plant, reaching the values ranging from 0.36 in roots up to 4.05 in their aboveground parts. In the latter, the higher doses of diesel fuel and engine oil led to the lowering of the value of K/(Ca + Mg) ratio. At the same time, almost all of the analysed PDSs caused the increase in the value of Ca/Mg ratio in the aboveground parts. The above changes were principally conditioned upon the increase in calcium content, with only slight changes in the magnesium contents in these parts of plants. The value of N/S ratio can differ much between plant species as well between the particular parts of plant. Jamal et al. (2010) indicate the ratio of 15:1 as an optimum value. In our earlier studies, we obtained similar values in broad bean plants growing on soil contaminated with the same doses of PDSs (Rusin et al. 2017). However, in the roots and shoots of spring wheat, Matraszek et al. (2015) obtained the values of this parameter ranging from 0.90 to 4.40. In the studies that we carried out, this parameter also attained low values—from 1.72 in aboveground parts, 3.84 in roots, and up to 4.62 in grain, which was principally associated with the relatively low nitrogen content. Under the effects of diesel fuel and the higher dose of petrol, the value of this parameter was slightly elevated, which could suggest the improvement in the condition of host plant. It was not, however, reflected in the developmental parameters of aphids.

The next factor that could affect the bionomics of *R. padi* aphids are the toxic substances that get into plants from contaminated soil. The contamination of soil with PDSs leads to the increase in the content of heavy metals in soil (Okonokhua et al. 2007; Ujowundu et al. 2011; Wyszkowski and Sivitskaya 2012), and many authors emphasise that the presence of heavy metals in soil can have a very great effect on the development of aphids on the plants, associated with the migration of harmful compounds to the aboveground parts of plants, which are the principal place of feeding by aphids (Görür 2009; Gospodarek 2012). Moreover, Merrington et al. (1997) found that aphids accumulate more toxic metals in the bodies than their host plants in the tissues. Görür (2007) demonstrated that the increase in lead content, and, to a lesser extent, the copper in the host plant contributes to the decrease in fecundity of females, intrinsic growth rate of the population as well as to the lengthening the pre-reproductive period in cabbage aphid (*Brevicoryne brassicae* L.). Also in our experiment, PDSs generally contributed to the increased contents of these heavy metals in the aboveground parts of plants, which is a fact that can partly explain the lowered values of developmental parameters of *R. padi*.

Gospodarek (2012) demonstrated that in the conditions of soil polluted with heavy metals, the length of pre-reproductive period in black bean aphid could extend to12.2 days (i.e. more than 5 days longer than in control group) and depended on the kind of metal and on the generation of the analysed pest. In our experiment, the higher doses of PDSs also caused the lengthening of the pre-reproductive period in the first generation of bird cherry-oat aphid by 0.8–1.6 days; however, it did not affect the studied parameter in the subsequent generations of the pest.

Crawford et al. (1995) found that the increased cadmium content in soil (and therefore its increased content in plants) contributed to the lengthening of the pre-reproductive period in black bean aphid from 8.1 to 10.1 days. In our experiment, it was only engine oil and petrol in the dose of 9 g kg^−1^ that caused a significant increase of cadmium content in the aboveground parts of plants, without significantly changing the length of pre-reproductive period. It should, nevertheless, be noted that the observed changes in the bionomy of *A. fabae* were associated with a much higher level of heavy metals in plant tissues than that noted in our experiment. Thus, the cadmium content in plant tissues during the studies by Crawford et al. (1995) has increased a few dozen times after this element was supplied to the substrate; whereas, in our experiment, it rose by approx. 50%. However, the multiple regressions showed the interrelations between the Cd content in aboveground parts of plants and the life span, fecundity, and intrinsic growth rate of aphids in their first generation.

Engine oil in a higher dose and diesel fuel in a lower dose contributed to the increase in the content of copper in aboveground parts of wheat by approx. 30–40%. The twofold increase of the content of this element in the tissues of broad bean was associated with the shortening of development period in *A. fabae* by approx. 1.1 days and with the increase in reproductive rate by approx. 1.4 progeny per day, although it did not affect the longevity, reproductive period, and the number of progeny per aphid (Crawford et al. 1995). The changes in the developmental parameters of *R. padi* noted in our experiment reached much further, and the negative relationship between the Cu content and the fecundity and intrinsic growth rate was found in the second generation of the pest.

The element whose content in aboveground parts of wheat increased clearly under the effect of higher doses of PDSs (and, in the case of petrol, also under the effect of lower dose) was manganese. A similar increase in its content, but only under the effect of diesel fuel, was noted in the leaves and shoots of broad bean (Rusin et al. 2017). The negative effect of this element on the developmental parameters of *R. padi* was demonstrated in the first generation. In available publications, there is no data on the effect of this element on the developmental parameters in aphids.

The negative relationships between the contents of both some nutrients and heavy metals and the life history traits of particular generations of *R. padi* may indicate a synergistic effect resulting from both the worsened nutritive value of host plant and from the transfer of harmful substances from the soils via plant to aphids, but it is too early to reach such conclusion.

## Conclusions

1. All of the applied petroleum-derived substances have a generally adverse effect on the developmental parameters in bird cherry-oat aphid, resulting in the decrease of its fecundity, shortening its average life span, and most often also causing the lowering of the population intrinsic growth rate.
2. PDSs caused the limitation of growth in wheat plants; whereas, changes in nutrient contents and heavy metals depended on the part of the plant analysed, the substance applied, and on its dose. The negative relationships between the contents of both some macro-elements (Ca, K, P) and heavy metals (Mn, Cd, Cu, and Zn) and the developmental parameters of particular generations of *R. padi* were observed.
3. Obtained results indicate that soil contamination with PDSs entails far-reaching changes in organisms indirectly exposed to these pollutants, i.e. herbivores, and as a result, it may also adversely affect the further links of the food chain, i.e. for predators and parasitoids.
4. The high susceptibility of *R. padi* to the presence of PDSs in the substrate for the host plant should be emphasised—the clear-cut changes in the life span and fecundity, with relatively small changes in the chemical composition of the plant, constitute an evident indication that the developmental parameters of aphids have the potential for the use as bio-indicator to evaluate the state of the environment contaminated by PDSs.
